# Supplementary material for: A Novel Effector Protein SsERP1 Inhibits Plant Ethylene Signaling to Promote Sclerotinia sclerotiorum Infection
Source: J Fungi (Basel). 2021 Oct 1;7(10):825. doi: 10.3390/jof7100825 (PMC8537369; doi:10.3390/jof7100825)
Supplement: Supplementary file 1 [file jof-07-00825-s001.zip › jof-1385941-supplementary.pdf]

Supplemental Material

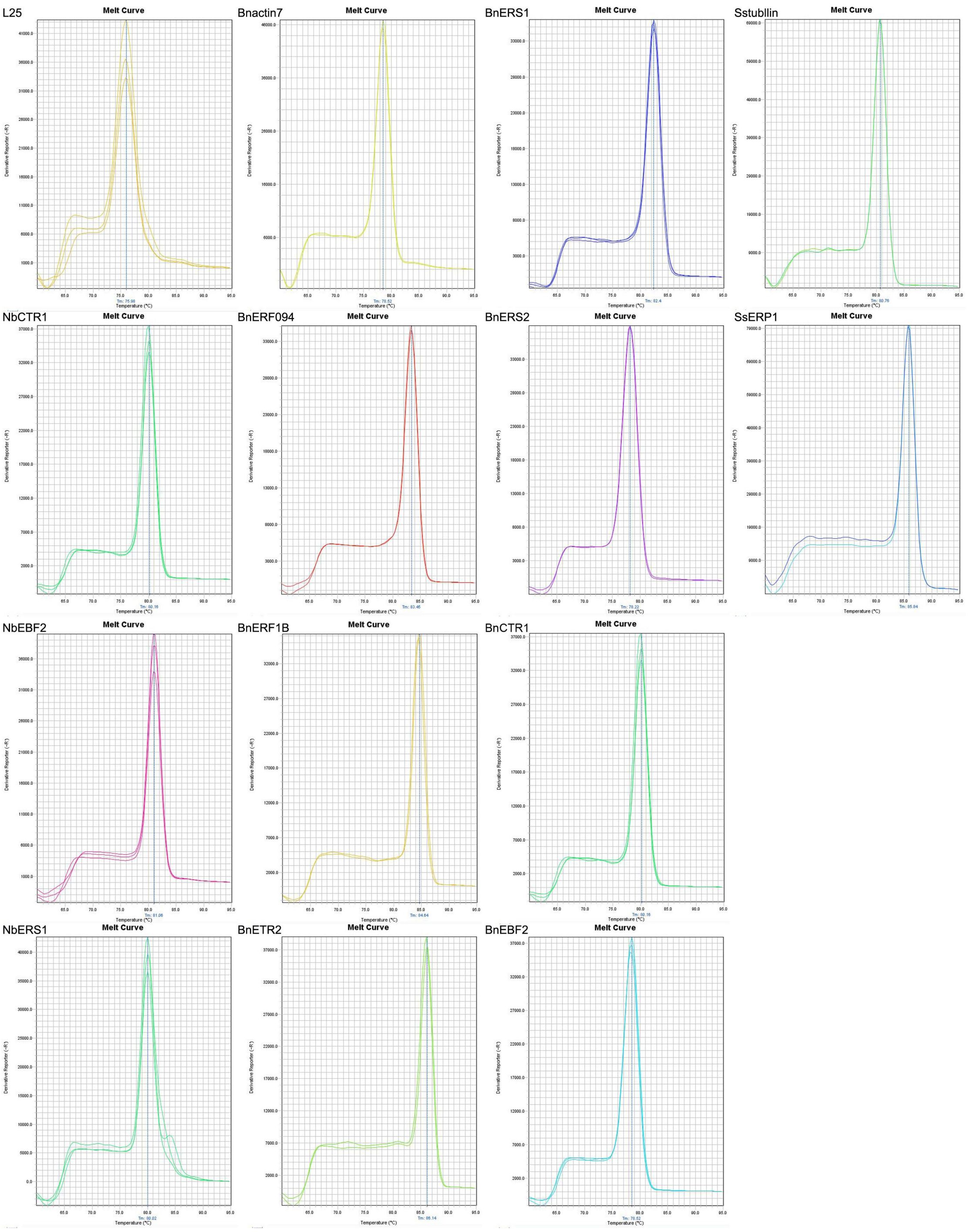

Figure S1. Melting curves of genes detected by qPCR in this study.

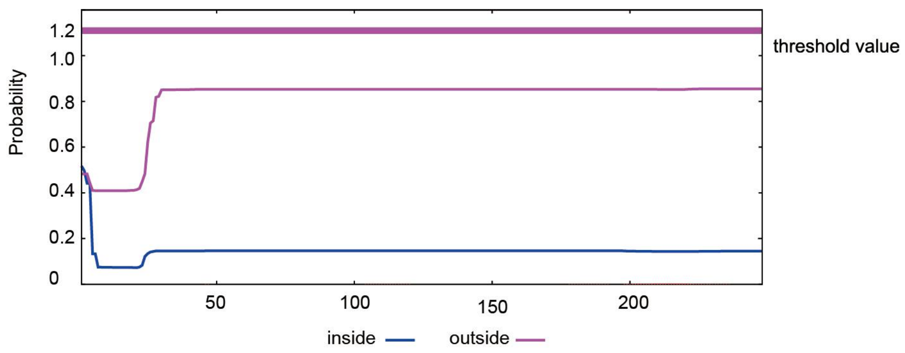

Figure S2. SsERP1 does not contain transmembrane domains

SsERP1 protein sequence was subjected to TMHMM(2.0) analysis for transmembrane domain identification.

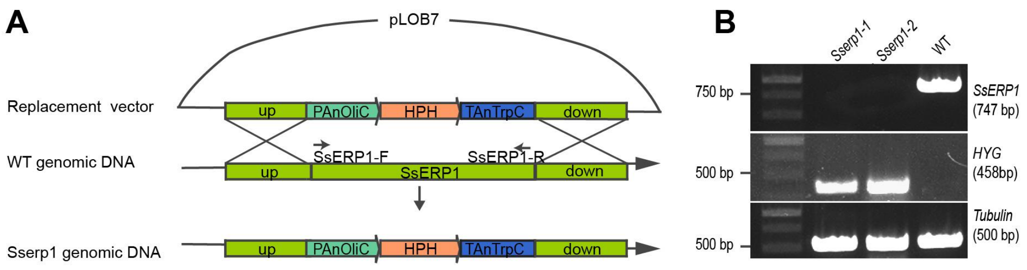

Figure S3. Identification of *SsERP1* knockout mutants

A, Schematic representation of homologous recombination-mediated deletion of *SsERP1* gene in *S. sclerotiorum*. "up" and "down" denote the 5' and 3' flanking regions of *SsERP1*, respectively. *PAnOliC*, *HPH*, and *TAnTrpC* represent the *AnOliC* promoter, the hygromycin resistance gene, and the *TAnTrpC* terminator, respectively.

B, Identification of *Sserp1* by PCR analysis. The primers *SsERP1*-F and *SsERP1*-R (as shown in Figure S3a) were used to verify the complete deletion of *SsERP1* gene in *Sserp1-1 and Sserp1-2* mutant strains, the primers for hygromycin gene(HYG) were used to verify the insertion of hygromycin gene in the genome of *S. sclerotiorum*, and the amplification using the primers *Tubulin* RT-F and *Tubulin* RT-R served as control for the quality of the *S. sclerotiorum* DNAs. Primer sequences are listed in supplementary table S1.

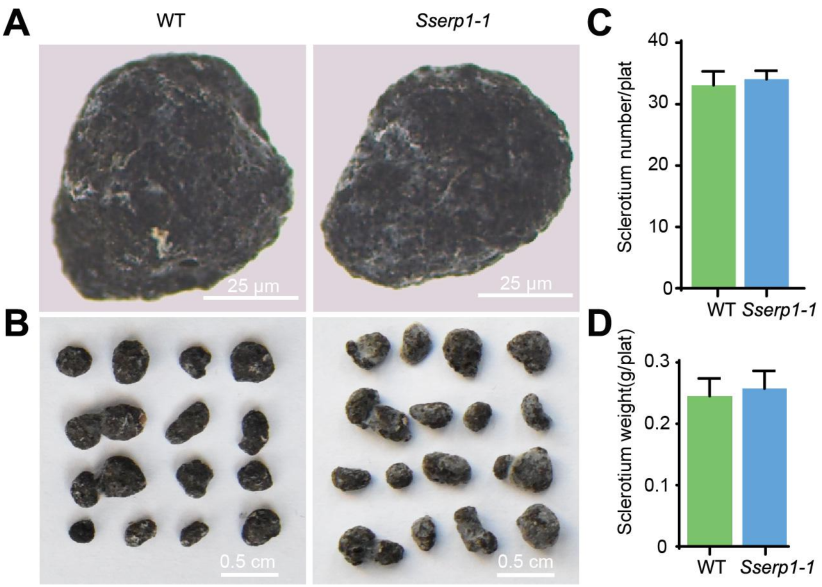

Figure S4. SsERP1 has no obvious impact on sclerotium formation

A and B, The morphology of sclerotia formed by *Sserp1* mutant (*Sserp1-1*) and wildtype strain (WT). C and D, the number and weight of sclerotia formed by *Sserp1-1* and WT per plate, respectively. Data are presented as mean ± SD, n=4. * denotes $p<0.05$ (one way ANOVA followed by Duncan's test).

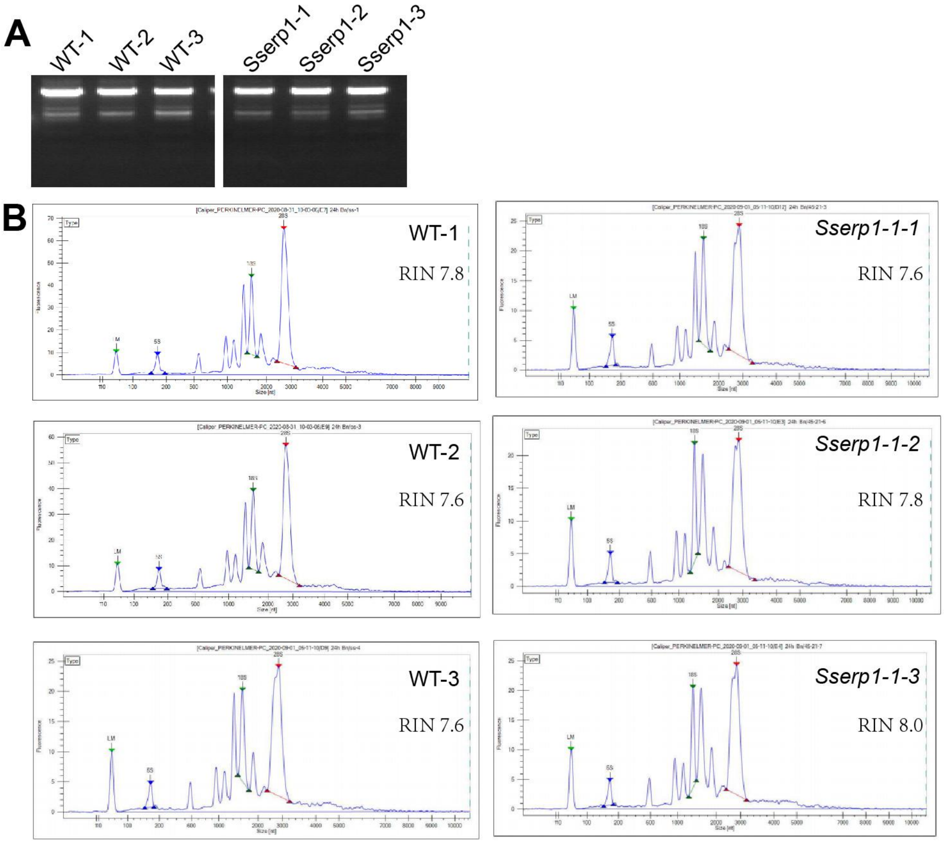

Figure S5. Quality check of RNA samples for transcriptome

A, Electropherograms presenting the RNA bands in agarose gels. B, RNA integrity assessed using the RNA Nano 6000 Assay Kit of the Agilent Bioanalyzer 2100 system.

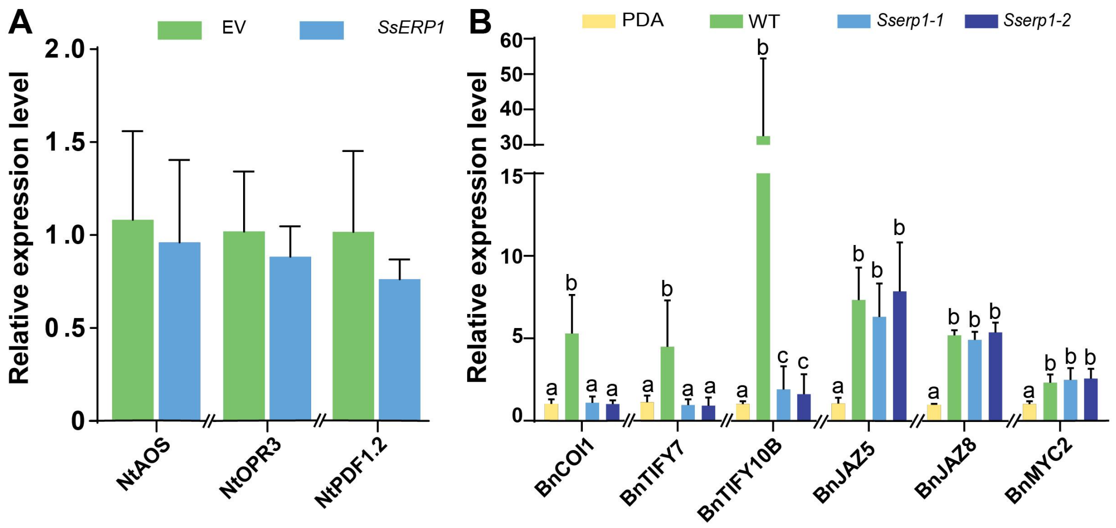

Figure S6. *SsERP1* did not affect the expression of the majority of jasmonate pathway genes

A, Tobacco leaves infiltrated with *Agrobacterium* containing *pTRV2-SsERP1* (*SsERP1*), or *pTRV2* empty vector (*EV*) as control, were collected for qPCR analysis 4 days after infiltration. Data are shown as mean ± SD, n=3.

B, Rapeseed leaves were inoculated with mycelium agar plugs of *Sserp1* mutants (*Sserp1-1* and *Sserp1-2*) or the wild-type strain (WT), or mock-treated with PDA medium (PDA), and leaves were collected for qPCR analysis 24 hours post inoculation. Data are shown as mean ± SD, n=3. ($p<0.05$, one-way ANOVA followed by Duncan's test).

A

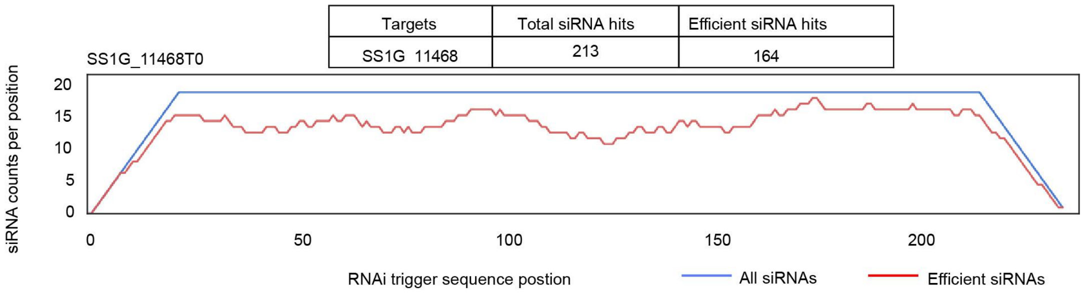

B

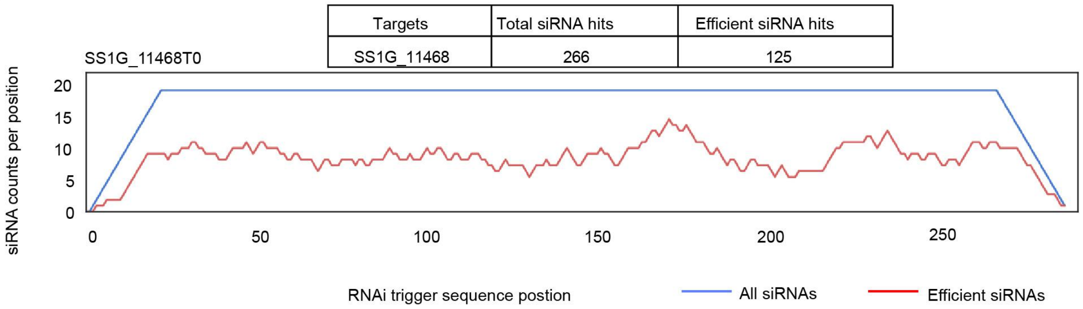

Figure S7. Target site prediction for the SsERP1A1 and SsERP1A2 dsRNAs

The target sites of SsERP1A1 (A) and SsERP1A2 (B) in S.sclerotiorum and tobacco were predicted using sifi21software. SsERP1 (SS1G_11468) was predicted to be the only target gene of SsERP1A1 and SsERP1A2, without potential off-target sites. The blue line denotes the number of total siRNA hits, and the red line denotes the number of efficient siRNA hits.

Table S1. Expression of ethylene biosynthesis pathway genes in *B.napus* leaves inoculated with *Sserp1* mutants or WT strains.

B. napus gene ID	*Sserp1-1* FPKM	*Sserp1-2* FPKM	*Sserp1-3* FPKM	WT-1 FPKM	WT-2 FPKM	WT-3 FPKM	FDR	log2FC	Differentially expressed	*A.thaliana* homologous gene name	*A.thaliana* homologous gene ID
BnC08g0877100	2.79	3.80	2.58	1.61	3.09	2.38	0.55	0.36	no	AtACO1	AT2G19590
BnA09g0345000	1.40	1.12	0.68	0.84	1.48	1.13	0.97	-0.05	no	AtACO1	AT2G19590
BnC04g0647190	2.09	12.18	2.53	3.23	7.94	13.07	0.87	-0.18	no	AtACO2	AT1G62380
BnA09g0348960	2711.41	2449.34	2473.47	1862.87	2263.99	2802.80	0.75	0.17	no	AtACO2	AT1G62380
BnC08g0873860	2595.71	1965.38	2207.54	1949.79	2042.17	2473.52	0.86	0.10	no	AtACO2	AT1G62380
BnC05g0688270	97.88	93.71	121.40	175.02	127.86	175.46	0.06	-0.52	no	AtACO4	AT1G05010
BnA09g0342600	85.18	89.73	97.85	153.41	118.01	164.75	0.02	-0.59	no	AtACO4	AT1G05010
BnC02g0499400	1.14	1.11	1.30	2.10	1.69	1.55	0.45	-0.43	no	AtACO5	AT1G77330
BnA08g0317300	1.14	1.23	1.57	1.98	2.21	1.30	0.55	-0.34	no	AtACS1	AT3G61510
BnC08g0870060	10.66	5.23	7.78	13.78	10.25	10.87	0.32	-0.43	no	AtACS1	AT3G61510
BnA10g0398630	34.22	34.26	42.26	72.14	60.54	70.18	0.00	-0.77	no	AtACS2	AT1G01480
BnC05g0685320	20.17	17.34	23.09	26.91	28.25	30.88	0.12	-0.42	no	AtACS2	AT1G01480
BnA01g0015210	24.73	27.44	26.67	32.66	32.34	34.48	0.27	-0.27	no	AtACS4	AT4G23850
BnC01g0441580	93.72	96.26	93.36	104.88	106.67	118.31	0.63	-0.17	no	AtACS4	AT4G23850
BnUnng0956780	60.09	55.37	64.53	52.19	59.77	78.15	0.97	-0.03	no	AtACS4	AT4G23850
BnA03g0133410	7.49	8.47	6.51	6.47	8.95	6.77	0.91	0.07	no	AtACS6	AT3G05970
BnC04g0668560	1.06	2.25	1.22	2.42	1.78	2.16	0.68	-0.33	no	AtACS6	AT3G05970
BnC03g0576990	30.25	33.24	31.63	28.33	31.47	32.64	0.86	0.09	no	AtACS6	AT3G05970
BnA08g0322430	2.10	2.24	1.95	0.37	2.00	0.97	0.21	0.67	no	AtACS8	AT4G37770
BnUnng0948710	2.33	2.62	3.25	2.55	2.35	2.10	0.65	0.25	no	AtACS10	AT1G62960

BnA09g0346430	1.13	1.80	1.37	1.39	1.33	0.98	0.77	0.22	no	AtACS10	AT1G62960
BnC02g0488130	2.89	3.72	2.76	2.68	3.41	2.18	0.71	0.22	no	AtACS12	AT5G51690
BnA02g0063590	5.37	4.74	5.43	4.68	4.87	3.36	0.44	0.30	no	AtACS12	AT5G51690

B.napus leaves were inoculated with mycelium agar plugs of *Sserp1-1* or the wild-type strain, and leaves were collected for transcriptome analysis 24 hours post inoculation.

Table S2. Expression of JA pathway genes in *B.napus* leaves inoculated with *Sserp1* mutants or WT strains.

B. napus Gene ID	*Sserp1-1* FPKM	*Sserp1-2* FPKM	*Sserp1-3* FPKM	WT-1 FPKM	WT-2 FPKM	WT-3 FPKM	DESeq2_FDR	DESeq2 log2FC	Differentially expressed	*A. thaliana* homologous gene name	*A. thaliana* homologous gene ID
BnUnng0942190	60.07	64.09	46.71	54.86	55.50	65.94	1.00	0.00	no	AtJAZ1	AT1G19180
BnC08g0877380	38.44	44.45	46.38	52.25	46.12	59.02	0.57	-0.22	no	AtJAZ1	AT1G19180
BnC08g0877370	25.28	26.20	27.72	33.27	21.22	39.80	0.77	-0.19	no	AtJAZ1	AT1G19180
BnC04g0679770	309.96	326.34	383.28	382.73	308.10	435.65	0.88	-0.09	no	AtJAZ1	AT1G19180
BnA09g0382530	87.13	103.93	101.52	103.31	102.94	103.95	0.95	-0.03	no	AtJAZ1	AT1G19180
BnA08g0328780	11.90	18.04	18.21	18.85	16.56	20.19	0.81	-0.15	no	AtJAZ1	AT1G19180
BnA06g0238550	155.59	155.00	183.45	204.79	168.56	225.41	0.61	-0.22	no	AtJAZ1	AT1G19180
BnC06g0771720	69.71	66.46	67.65	89.54	88.74	85.20	0.13	-0.31	no	AtJAZ2(TIFY10B)	AT1G74950
BnC02g0498350	20.96	18.32	21.00	31.57	24.31	30.34	0.13	-0.43	no	AtJAZ2(TIFY10B)	AT1G74950
BnA07g0299080	43.07	40.27	41.24	57.82	51.74	47.29	0.30	-0.27	no	AtJAZ2(TIFY10B)	AT1G74950
BnA07g0288510	7.01	9.06	5.52	16.61	13.89	10.48	0.01	-0.77	no	AtJAZ2(TIFY10B)	AT1G74950
BnC04g0635460	0.87	1.24	0.51	3.13	3.22	2.61	0.00	-1.24	down	AtJAZ2(TIFY10B)	AT1G74950
BnC08g0839710	27.18	25.69	22.54	18.73	25.60	28.45	0.90	0.09	no	AtJAZ3	AT3G17860
BnA05g0212810	2.23	4.14	0.52	2.36	1.19	0.58	0.59	0.43	no	AtJAZ3	AT3G17860
BnA01g0034500	3.96	4.13	3.20	3.06	3.55	3.08	0.64	0.24	no	AtJAZ3	AT3G17860
BnA01g0034490	4.91	4.89	5.29	6.13	6.43	7.28	0.43	-0.32	no	AtJAZ3	AT3G17860
BnC08g0878020	9.35	8.11	8.33	20.09	15.32	15.13	0.00	-0.85	no	AtJAZ5	AT1G17380
BnC08g0854020	92.11	93.27	88.22	181.19	147.72	200.69	0.00	-0.85	no	AtJAZ5	AT1G17380
BnA09g0383400	17.42	15.04	15.15	32.74	34.66	28.09	0.00	-0.90	no	AtJAZ5	AT1G17380
BnA08g0329850	113.14	113.57	131.91	189.62	162.92	206.14	0.01	-0.56	no	AtJAZ5	AT1G17380

BnC06g0769420	37.72	34.69	39.19	45.23	41.58	47.20	0.58	-0.20	no	AtJAZ6	AT1G72450
BnC02g0516000	94.08	102.14	109.08	113.53	97.83	132.97	0.82	-0.12	no	AtJAZ6	AT1G72450
BnA07g0296790	10.75	10.62	11.82	20.94	22.47	18.45	0.00	-0.80	no	AtJAZ6	AT1G72450
BnA03g0128880	101.15	104.60	102.88	134.99	97.24	154.55	0.55	-0.26	no	AtJAZ6	AT1G72450
BnC05g0711590	28.77	38.23	39.74	16.69	21.88	22.74	0.00	0.77	no	AtJAZ8	AT1G30135
BnC03g0602700	40.09	44.11	71.62	71.14	54.87	68.78	0.68	-0.24	no	AtJAZ8	AT1G30135
BnA09g0365320	40.53	46.58	47.59	23.84	26.40	33.88	0.01	0.66	no	AtJAZ8	AT1G30135
BnA08g0324340	27.64	38.52	44.50	61.47	41.01	54.76	0.30	-0.41	no	AtJAZ8	AT1G30135
BnC06g0767570	170.57	179.79	180.51	213.25	209.79	214.64	0.43	-0.21	no	AtJAZ9(TIFY7)	AT1G70700
BnC02g0495420	21.00	27.77	23.66	33.08	27.51	29.17	0.48	-0.25	no	AtJAZ9(TIFY7)	AT1G70700
BnA07g0290190	14.81	19.63	25.94	34.84	26.67	40.93	0.06	-0.63	no	AtJAZ9(TIFY7)	AT1G70700
BnA02g0068600	52.99	53.48	55.84	67.64	58.05	70.72	0.55	-0.22	no	AtJAZ9(TIFY7)	AT1G70700
BnC06g0759780	11.46	14.64	17.72	32.77	31.73	38.16	0.00	-1.07	down	AtJAZ9(TIFY7)	AT1G70700
BnC09g0928500	70.93	66.14	74.68	89.79	76.06	111.25	0.40	-0.32	no	AtJAZ10	AT5G13220
BnC07g0827940	5.90	2.76	5.33	3.05	5.40	3.24	0.76	0.25	no	AtJAZ10	AT5G13220
BnC03g0540620	23.25	30.62	48.87	61.80	41.46	63.61	0.17	-0.56	no	AtJAZ10	AT5G13220
BnA10g0414670	60.74	71.15	78.59	90.71	68.68	98.58	0.62	-0.23	no	AtJAZ10	AT5G13220
BnA03g0095680	9.14	11.40	19.17	29.28	17.01	31.18	0.05	-0.75	no	AtJAZ10	AT5G13220
BnA02g0051320	4.83	4.83	4.82	5.28	5.67	3.64	0.96	0.04	no	AtJAZ10	AT5G13220
BnC09g0920640	33.29	34.25	34.20	34.39	35.53	33.60	0.96	0.03	no	AtJAZ12	AT5G20900
BnA10g0407350	147.52	158.43	147.35	169.86	159.81	164.41	0.87	-0.07	no	AtJAZ12	AT5G20900
BnA02g0055990	82.02	88.49	102.55	96.16	104.08	100.15	0.87	-0.08	no	AtJAZ12	AT5G20900
BnUnng0988210	3.16	15.47	12.03	16.59	13.94	6.06	0.95	-0.09	no	MYC2	AT1G32640
BnUnng1011260	2.35	1.75	1.03	2.43	2.86	2.74	0.30	-0.49	no	MYC2	AT1G32640

BnC06g0737780	17.92	11.01	13.23	13.93	21.27	23.31	0.42	-0.37	no	MYC2	AT1G32640
BnC07g0804200	1.40	1.45	0.80	2.04	2.37	1.77	0.15	-0.58	no	MYC2	AT1G32640
BnA05g0200360	47.63	58.83	79.42	121.78	72.54	184.59	0.03	-0.82	no	AOS	AT5G42650
BnC02g0505610	68.76	75.38	87.65	133.91	98.57	166.26	0.02	-0.67	no	AOS	AT5G42650
BnUnng0992950	43.56	51.96	56.60	73.46	49.94	138.20	0.49	-0.49	no	OPR3	AT2G06050
BnUnng1014220	29.36	30.91	37.97	86.70	61.13	62.53	0.00	-0.96	no	OPR3	AT2G06050
BnA08g0329820	0.18	0.37	0.19	0.39	0.35	1.00	0.17	-0.75	no	LOX3	AT1G17420

B.napus leaves were inoculated with mycelium agar plugs of *Sserp1-1* or the wild-type strain, and leaves were collected for transcriptome analysis 24 hours post inoculation.

Table S3. Primers for vector construction.

Primer name	Sequence
SsERP1(PSUC2)OS-F	TCGGAATTTTAATTAAATGCGTTATACTATC
SsERP1SP(PSUC2)OS-R	CACTATAGGGAGAACGGCGGCAACGAATGG
SsERP1(pGJ186)-Sac1F	AGAGAGCTCATGCGTTATACTATCGTCATC
SsERP1(pGJ186)-Mlu1R	AGAACGCGTTAGCACGGAACTTGGCACCG
SsERP1DSP(pGJ186)-Sac1F	AGAGAGCTCATGCATGGCAAGATCGCAGTCTTG
SsERP1DSP(pTRV2)BamH1-F	CGGGATCCCATGGCAAGATCGCAGTCTTGT
SsERP1(pTRV2)BamH1-F	CGGGATCCATGCGTTATACTATCGTCATC
SsERP1(pLOB7)up-Sac1F	GCCATATTGATTTCCATCTC
SsERP1(pLOB7)up-Sal1R	ATTTGCTGTTGGGGTTGGTTG
SsERP1(pLOB7)down-Asc1F	TTTTTCAGAATTCAAGATTACGG
SsERP1(pLOB7)down-Hind3R	CAACGGTAATGAAGATTGATAG

Table S4. Primers for *Sserp1* mutant identification.

**Primer name**	**Sequence**
SsERP1-F	ATGCGTTATACTATCGTCATC
SsERP1-R	TTAAGCACGGAACTTGGCACC
HYG-F	ATGGCTGAACTCACCGCGAC
HYG-R	CATCCATAGCCTCCGCGACC
Tubulin-RT-F	GTTCATCTTCAAACCGGCCAATGT
Tubulin-RT-R	CAGTTGTTACCAGCACCGGATTGA

Table S5. Primers for dsRNA template amplification.

Primer name	Sequence
SsERP1A1	TAATACGACTCACTATAGGGCAACAGCAAGAATGCCGCTAC
SsERP1B1	TAATACGACTCACTATAGGGAGTCCTCGTTGACGTTCTTGG
SsERP1A2	TAATACGACTCACTATAGGGCACTACTCAAGTACCAGGCAG
SsERP1B2	TAATACGACTCACTATAGGGTGCCATTACCAGCAGCAGCAT

Table S6. Primers for qPCR analysis.

Primer name	Sequence
SsTubulin-qPCR-F	TTGGATTTGCTCCTTTGACCAG
SsTubulin-qPCR-R	AGCGGCCATCATGTTCTTAGG
BnActin7-F	TCTTCCTCACGCTATCCTCCG
BnActin7-R	AGCCGTCTCCAGCTCTTGC
L25-F	CCCCTCACCACAGAGTCTGC
L25-R	AAGGGTGTTGTTGTCCTCAATCTT
BnERF094q-162-F	AACGAGGAAAGGGATAAGAGTG
BnERF094q-162-R	TTCACATTCTCCATGTTCCAGA
BnERF1Bq-271-F	ATGCTTCTCTACGGACTAATCG
BnERF1Bq-271-R	CCGGAAAATTTAATATCGCCGA
BnETR2q-178-F	TGTTTGTAAGAAAGTGGTGCAG
BnETR2q-178-R	AGAATCAGAATGAGGGTGAAGG
BnCTR1q-142-F	CTATCTCCATAATCGCAACCCT
BnCTR1q-142-R	CGACTTTGATGAAAGGAAGGTG
BnEBF2q-86-F	AGTTTGAAACTTGAGGAATGCC
BnEBF2q-86-R	AAAGACTTCAACTTTACGCCAC
BnERS1q-137-F	GACACACTATCCTAAGGACCAC
BnERS1q-137-R	TCCGACTTGTATCTTATGGCTC
BnERS2q-80-F	AACAGACTGAGACTAGTTTGCA
BnERS2q-80-R	GTAGAGTATCGTGTGCCTATCC
NbCTR1q-186-F	TCAGGACAAACTGACCGAAATA
NbCTR1q-186-R	TAGTTGACTTCCTCTCTCGAGA
NbEBF2q-214-F	CGTTGGTAGCGATAACATGAAG
NbEBF2q-214-R	CAACAAGTAGGACCAGACTTGA
NbERS1q-175-F	CTATTTTTCCATTCCGCTGGAG
NbERS1q-175-R	TATAGTCATAACCACAGCGACC
NbAOS-like-qF	CACACTCAATTAGCTCAAGAG
NbAOS-like -qR	GGATTCAATCACCATATCTCG
NbPDF1.2-qF	GACCAACGACAATTGCAGAGG
NbPDF1.2-qR	GTCAAACAGACGGTGGCACA
NbOPR3-qF	GTGGAGCTGCACCAATATCT
NbOPR3-qR	TGCCTTGCGATAATCTTCAACC
